# Enrichment of the Cancer Stem Phenotype in Sphere Cultures of Prostate Cancer Cell Lines Occurs through Activation of Developmental Pathways Mediated by the Transcriptional Regulator ΔNp63α

**DOI:** 10.1371/journal.pone.0130118

**Published:** 2015-06-25

**Authors:** Roberto Portillo-Lara, Mario Moisés Alvarez

**Affiliations:** 1 Centro de Biotecnología-FEMSA, Tecnológico de Monterrey, Monterrey, Nuevo León, México; 2 Harvard-MIT Health Sciences and Technology, Brigham and Women’s Hospital, Cambridge, Massachusetts, United States of America; University of Colorado Denver, UNITED STATES

## Abstract

**Background:**

Cancer stem cells (CSC) drive prostate cancer tumor survival and metastasis. Nevertheless, the development of specific therapies against CSCs is hindered by the scarcity of these cells in prostate tissues. Suspension culture systems have been reported to enrich CSCs in primary cultures and cell lines. However, the molecular mechanisms underlying this phenomenon have not been fully explored.

**Methodology/Principal Findings:**

We describe a prostasphere assay for the enrichment of CD133^+^ CSCs in four commercial PCa cell lines: 22Rv1, DU145, LNCaP, and PC3. Overexpression of CD133, as determined by flow cytometric analysis, correlated with an increased clonogenic, chemoresistant, and invasive potential in vitro. This phenotype is concordant to that of CSCs in vivo. Gene expression profiling was then carried out using the Cancer Reference panel and the nCounter system from NanoString Technologies. This analysis revealed several upregulated transcripts that can be further explored as potential diagnostic markers or therapeutic targets. Furthermore, functional annotation analysis suggests that ΔNp63α modulates the activation of developmental pathways responsible for the increased stem identity of cells growing in suspension cultures.

**Conclusions/Significance:**

We conclude that profiling the genetic mechanisms involved in CSC enrichment will help us to better understand the molecular pathways that underlie CSC pathophysiology. This platform can be readily adapted to enrich and assay actual patient samples, in order to design patient-specific therapies that are aimed particularly against CSCs.

## Introduction

Prostate cancer (PCa) is the second most common malignancy in man worldwide, and yet its etiology is still largely unresolved. As is the case in other epithelial tissues, cellular homeostasis in the adult prostate is maintained through hierarchically organized cells with different proliferative potentials [1]. Somatic SCs located at the apex of this hierarchy exhibit some unique characteristics, including: the ability to self-renew and differentiate along several cell lineages, localized growth in specialized physiological microenvironments (niches), and although they are normally quiescent, they display a remarkable proliferative potential [2]. The hierarchical stem cell (SC) model of carcinogenesis holds that PCa originates through alterations of genetic and epigenetic factors that regulate the proliferation of normal SCs [3]. These aberrantly expressed pathways ultimately lead to the transformation of normal SCs into malignant cancer stem cells (CSCs). CSCs retain some of the characteristics associated with their non-malignant counterparts, and are thought to be responsible for tumor initiation, progression and relapse, as well as metastatic disease.

CSCs are also thought to be responsible for the development of resistance to conventional therapies [4,5]. Traditional radio- and chemo-therapeutic agents are conceived under the notion that all cells within a tumor are phenotypically equal. However, CSCs rely on intrinsic mechanisms that render them comparatively more resilient than terminally differentiated cells, including their slowly proliferating nature, high expression of ATP-binding cassette (ABC) membrane transporters, and resistance to DNA damage and oxidative stress [6]. Therefore, CSC-specific therapies have the potential to eradicate the disease at its origin, and to spare non-tumorigenic SCs, thereby minimizing the adverse effects associated with otherwise nondiscriminating treatments.

Current methodologies for the isolation and study of CSCs are based on the expression of surface markers first identified in normal prostate SCs (CD44^+^/α_2_β_1_^hi^/CD133^+^) [7]. CD133 in particular, has been widely utilized as a biomarker for the isolation of cells that exhibit a stem-like phenotype in a variety of normal and malignant tissues [8,9]. The isolated study of CSCs allows the analysis of molecular mechanisms responsible for malignant cell survival, separately from those occurring in terminally differentiated tumor cells or in non-tumorigenic SC populations. Nevertheless, the development of therapeutic strategies based on CSC-targeting has been limited due to the lack of suitable experimental models.

Human primary tumor samples (PTS) are considered to be the most accurate representation of the tumor in vivo. However, the complexity of the physiological context often influences the interpretation of the results. In contrast, human cancer cell lines (CCLs) are more dynamic tools that can be used to dissect the many different molecular processes involved in carcinogenesis. Although PTS are still favored over CCLs, their highly restricted access makes in vitro models the “go-to” choice for many research groups worldwide.

Recent studies have demonstrated that CCLs are similar to in vivo malignant tumors, as they also consist of organizational hierarchies with different degrees of differentiation [10–12]. Flow cytometry analysis of PCa cell lines indicates that even after being propagated for years, they still contain detectable numbers of stem-like cells, albeit often at extremely low percentages. Pfeiffer et al. reported that CD133^+^ cells within the DU145 cell line account for approximately 0.01% of the total population, whereas DuCaP, LAPC-4, LNCaP, PC3, and 22Rv1 yield no detectable CD133 expression when analyzed via fluorescence activated cell sorting (FACS) [13]. Thus, although CCLs represent a highly accessible and practical alternative to PTSs, isolation of CSCs from this source remains difficult due to the small numbers of undifferentiated cells contained in commercial cell lines.

Suspension culture systems are increasingly being used to enrich undifferentiated cell populations in PTSs and CCLs. Cells propagated using non-adherent substrates and serum-free media grow as three-dimensional spheroid cell clusters (tumorspheres) that promote the proliferation of progenitor cell phenotypes [14,15]. This is a complex phenomenon in which rare undifferentiated cells are stimulated to engage in symmetrical division to expand the SC compartment. Although several variations of the tumorsphere assay have been reported, the genetic mechanisms that trigger stem cell proliferation in spheroid cultures have not been fully explored.

Modern gene expression analysis tools allow the study of transcriptional alterations with increased sensitivity and specificity, compared to conventional microarray-based approaches that are limited by major technological challenges [16]. For example, the nCounter Nanostring system delivers digital readouts of each molecule of a target mRNA, using minimum amounts of starting material, and without the need for cDNA synthesis [17]. Briefly, this system makes use of pairs of capture and reporter probes that are tailored to each mRNA target sequence. After hybridization, the transcript/probe complexes are immobilized, aligned in an electrical field, identified, and enumerated based on color-coded tags that are coupled to the reporter probe.

Coupling the dynamics of in vitro models with high-throughput gene expression profiling could aid in the development of more effective CSC-targeted therapeutic strategies. Here, we have adapted a tumorsphere culture protocol for the effective enrichment of CD133^+^ CSCs in four commercial PCa cell lines: 22Rv1, DU145, LNCaP, and PC3. All cell lines were able to form highly proliferating and self-renewing spheres when plated in the serum-free, anchorage-independent conditions that we used. We demonstrate that CSC-associated features (e.g., CD133 expression, as well as increased proliferative, invasive, and chemoresistant potential) were significantly enriched in all prostasphere cultures. Gene expression profiling of parental and enriched cultures using the nCounter NanoString system revealed several upregulated transcripts that participate in the establishment of the malignant stem phenotype. Furthermore, functional annotation suggests that the CSC-enrichment observed in prostasphere cultures occurs via the activation of developmental pathways modulated in part by the transcriptional regulator ΔNp63α.

## Materials and Methods

### Cell lines and culture protocols

All cell lines were purchased directly from ATCC. Cells were used at the following passage numbers: 22Rv1, passage 11; DU145, passage 16; LNCaP, passage 13; PC3, passage 18. For gene expression profiling experiments, PC3 cells were grown in 6-well ultra-low attachment culture plates with EpiGRO human Prostate Complete Media Kit (hPCM, Millipore). For the rest of the experiments, the four cell lines were propagated using three different culture protocols: a) DMEM-FBS: monolayer cultures in DMEM-F12 (Gibco) supplemented with 5% fetal bovine serum (FBS, Invitrogen) and 100 U/100 μg/ml penicillin-streptomycin (Gibco); b) DMEM-PLUS: non-adherent sphere (prostasphere, PS) cultures grown in 6-well ultra-low attachment culture plates (Corning) with DMEM-F12 supplemented with 20 ng/μl hEGF (Gibco), 20 ng/μl bFGF (Gibco), 1x B27 without vitamin A (Invitrogen), 1x Insulin-Transferrin-Selenium A (Invitrogen) and 100 U/100 μg/ml penicillin-streptomycin; and c) hPCM-PLUS: sphere cultures in hPCM, further supplemented with 20 ng/μl hEGF and 20 ng/μl bFGF. Monolayer cultures were maintained at a maximum confluence level of 80% and culture medium was replaced every 48 hours. Prostasphere cultures were seeded at a density of 2x10^4^ cells/ml and culture media was fully replaced every 96 hours on day 4, and 8. Parental and prostasphere cultures were propagated for 12 days at 37°C at 95% relative humidity in a 5% CO_2_ and 95% air atmosphere. At the end of the culture protocol, cells growing in monolayer and suspension cultures were enzymatically dissociated using 0.05% trypsin-EDTA solution (Gibco) and StemPro Accutase (Gibco), respectively, according to instructions from the manufacturer.

### Immunofluorescent labeling, cell sorting, and flow cytometry analysis

Dissociated cells were fluorescently labeled according to the manufacturer’s instructions using two monoclonal antibodies: Anti-Human CD133/2 (293C3)-PE (Miltenyi Biotec) and Anti-Human CD44 PE-Cyanine5 (eBioscience). Magnetic-activated cell sorting (MACS) was performed using Anti-Phycoerythrin microbeads (Miltenyi Biotec) and the CD133/2 (293C3)-PE monoclonal antibody according to the manufacturer’s instructions. Flow cytometry analysis was performed with a FACSCanto II flow cytometer with a 488-nm laser. A minimum of 10^4^ events was recorded for each reading. Anti-Mouse IgG2b-PE isotype control (Miltenyi) and unstained cells were used as controls. Experiments were carried out in triplicate and repeated three times.

#### nCounter NanoString gene expression profiling

Total RNA was extracted from PC3 prostaspheres and purified using the RNeasy Mini or Micro kits depending on the amount of starting material (Qiagen) according to the manufacturer’s instructions. The integrity of purified RNA samples was evaluated using a NanoDrop ND-1000 spectrophotometer at 260/280 nm. Detection of mRNA transcripts was then carried out in multiplexed hybridization reactions using the NanoString nCounter Analysis System. Two different nCounter Gene Expression CodeSets were used for the analysis: the GX Human Cancer Reference Kit consisting of 230 cancer-related genes, and the custom-made ITESM CodeSet consisting of 14 PCa-related genes (NanoString Technologies). Data acquisition and normalization was carried out using the nSolver Analysis software version 2.0 (NanoString Technologies). Average fold changes (ratios, n = 3) of each gene were calculated from normalized data and ranked using Microsoft Excel. Genes with a fold change > 1.5 were selected for further analysis (Table 1). General definitions for each gene were retrieved from the GeneCards encyclopedia webpage (www.genecards.org) [18].

**Table 1:** Upregulated genes in CD133^+^ sorted and unsorted prostasphere cells.

Prostasphere vs. Parental				CD133+ vs. CD133-				CD133+ vs. Parental	
Symbol	Ratio	Symbol	Ratio	Symbol	Ratio	Symbol	Ratio	Symbol	Ratio
*GAS1*	5.36	*CCND3*	1.64	*CSF3*	20.13	*CDK6*	1.93	*IGFBP3*	11.30
*IGFBP3*	5.24	*IFNGR1*	1.63	*TERT*	7.76	*WFDC2*	1.93	*CSF3*	9.01
*FGFR2*	3.91	*ERBB2*	1.61	*KLK3*	7.23	*GATA1*	1.93	*IL1A*	6.03
*IL1A*	3.46	*ITGB1*	1.61	*IL1A*	5.10	*MUC1*	1.90	*BCL2A1*	4.11
*MMP9*	3.13	*ABCB1*	1.58	*IGFBP3*	4.64	*PTPRG*	1.87	*EGR1*	4.00
*CASP10*	2.81	*MMP14*	1.58	*CCND2*	3.99	*BCL3*	1.87	*NOTCH3*	3.49
*CTGF*	2.66	*NOTCH3*	1.57	*BCL2A1*	3.64	*TOP2A*	1.86	*TIMP3*	3.34
*FOLR1*	2.66	*MPL*	1.55	*FRZB*	3.64	*E2F1*	1.86	*SHH*	3.25
*MYCL1*	2.43	*MCL1*	1.54	*PDGFRA*	3.49	*NUMA1*	1.83	*CTGF*	2.80
*STAT1*	2.25	*CAV1*	1.53	*PDGFRB*	3.07	*NF1*	1.83	*NANOG*	2.59
*BCL2*	2.16	*PIM1*	1.51	*IL6*	2.83	*MST1R*	1.82	*PDGFRA*	2.49
*FAS*	2.14	*MAPK10*	1.51	*KDR*	2.70	*ABL1*	1.79	*MMP9*	2.48
*SOX2*	2.13			*NANOG*	2.56	*HMMR*	1.79	*PDGFRB*	2.48
*PDGFRB*	2.12			*PTK7*	2.55	*LAMB1*	1.79	*PTPRG*	2.43
*CXCL9*	2.07			*MMP2*	2.51	*PROM1*	1.77	*KLK3*	2.18
*TIMP3*	2.05			*LMO1*	2.47	*CYP1A1*	1.74	*FRZB*	2.15
*LYN*	2.04			*NOTCH3*	2.39	*RARA*	1.72	*ATM*	2.07
*FRZB*	2.04			*NOTCH1*	2.38	*MLL*	1.70	*WFDC2*	2.07
*MYCN*	1.98			*FGFR4*	2.33	*FANCG*	1.68	*ERBB2*	1.91
*STAT3*	1.97			*MYCL1*	2.33	*RAD54L*	1.67	*PTK7*	1.91
*PLA2G2A*	1.97			*REL*	2.32	*PIM1*	1.66	*PLA2G2A*	1.83
*ESR1*	1.96			*ATM*	2.27	*ESR1*	1.65	*KDR*	1.77
*CDKN1A*	1.94			*WNT10B*	2.23	*BRCA2*	1.61	*TERT*	1.75
*ERBB4*	1.90			*FGFR1*	2.21	*BCR*	1.61	*SERPINE1*	1.66
*CD34*	1.88			*FAT1*	2.17	*PTGS2*	1.60	*PIM1*	1.65
*MUC1*	1.86			*COL1A1*	2.15	*EGFR*	1.59	*EGFR*	1.63
*BCL2L1*	1.86			*MAP3K8*	2.15	*LIF*	1.59	*TP73L*	1.59
*TP73L*	1.85			*SHH*	2.13	*MET*	1.58	*WNT4*	1.58
*WT1*	1.82			*PLAT*	2.02	*ERBB3*	1.55	*COL1A1*	1.57
*TNFSF10*	1.80			*L1CAM*	2.00	*SOX2*	1.53	*MUC1*	1.55
*PDGFA*	1.76			*MYBL2*	1.97	*WT1*	1.52	*CD34*	1.50
*NGFR*	1.74			*SIAH1*	1.96	*ERBB2*	1.51		
*EGFR*	1.71			*CDH11*	1.95	*TP73L*	1.51		
*MMP2*	1.70			*CEBPA*	1.95	*AKT2*	1.50		
*FYN*	1.64			*TYRO3*	1.94				

### Prostasphere self-renewal assay

The percentage of cells in enriched cultures capable of generating new prostaspheres was determined by plating cells at a density of 1x10^3^ cells/ml in the hPCM-PLUS media. On day 10 of culture, the spheres (>100 μm) were scored, dissociated, and sub-cultured under the same conditions. This process was repeated twice in order to obtain 2^nd^ and 3^rd^ generation prostaspheres at day 20 and 30, respectively. Sphere formation efficiency (SFE) was calculated as the number of prostaspheres generated by the number of cells seeded, multiplied by 100. Experiments were carried out in triplicate and repeated three times. Subsequent experiments were performed using first generation spheres only.

### Colony forming cell (CFC) assays

Suspension CFC assays were carried out using StemXVivo methylcellulose concentrate (R&D Systems) combined with DMEM-FBS without phenol red or hPCM-PLUS media. Briefly, 1x10^3^ cells were seeded in 35-mm petri dishes with semisolid culture medium. After 14 days, cultures were visualized under an inverted microscope and colonies larger than 100 μm were manually scored from an average of 5 random fields per dish. For adherent CFC assays, 1x10^3^ cells were plated in 35-mm tissue culture treated dishes with DMEM-FBS or hPCM-PLUS. After 14 days, cultures were fixed using 3.7% formaldehyde in PBS (1X) and stained with crystal violet stain solution (Fisher Scientific). Colonies consisting approximately of more than 50 cells were scored. Image analysis was performed using the ImageJ software (rsbweb.nih.gov/ij). Colony-forming efficiencies were calculated as the percentage of plated cells that were able to form clones. Suspension CFC experiments were performed in duplicate and repeated three times. Adherent CFC experiments were performed in triplicate and repeated three times.

### In vitro chemoresistance assay

Cell viability in response to the chemotherapeutic drug Docetaxel was assessed using the CellTiter 96 AQ_ueous_ One Solution Reagent (Promega). The basal drug sensitivity of reference cultures was established by plating 1x10^4^ cells from all four adherent PCa cell lines in each well of a 96-well plate in 100 μl DMEM-F12. After 24 hours, cells were incubated with 100 μl DMEM-F12 at a final concentration of 10, 5, 2.5, 1.25, and 0.625 μg/ml Docetaxel (Sigma-Aldrich). Subsequently, and based on the behavior of reference cultures exposed to increasing concentrations of Docetaxel, prostasphere cultures were assayed in a similar way. Briefly, 1x10^4^ monolayer cells, as well as CD133^+^-sorted and unsorted prostasphere cells were plated in 96-well plates at a final concentration of 5 and 10 μg/ml Docetaxel in 100 μl culture media. Cell viability was determined according to the manufacturer’s instructions. Each treatment was run in triplicate, and the experiment was repeated three times.

### In vitro basement membrane matrix invasion assay

The invasive potential of reference and enriched PCa cell lines was assessed using the Transwell assay. Briefly, 12 day-old cultures were dissociated and maintained in serum-free DMEM-F12 (for monolayer cultures) and serum-free DMEM-F12 without phenol red (for prostasphere cultures) for 24 hours. Starved cultures were then seeded in 200 μl serum-free media on 6.5 mm Transwell inserts with an 8-μm pore size PET membrane (Corning), coated with 50 μl Geltrex LDEV-free reduced growth factor basement membrane matrix (Gibco) at a density of 1x10^5^ cells per insert. A 650 μl volume of DMEM-FBS was added to the lower chamber as a chemoattractant. Cells were incubated at 37°C and allowed to invade through the matrix for 24 hours. Non-invading cells were carefully removed from the upper chamber using a cotton swab. Invading cells were fixed and stained using crystal violet stain solution. The number of migrated cells was calculated from images of five random x10 fields of view acquired with an Axiovert 200 inverted microscope. Images were analyzed using ImageJ software. The experiments were carried out in duplicate and repeated twice.

### Functional annotation clustering and Gene Ontology (GO) analysis

The DAVID Functional Annotation Tool [19] and the overrepresentation analysis tool of ConsensusPathDB [20] were used to identify over-represented signaling pathways using data from upregulated genes (fold change > 1.5) through functional enrichment analysis.

### Statistical Analysis

Results for continuous variables are expressed as means ± standard deviation (StdDev). Pairwise multiple comparisons were performed using a one-way analysis of variance (ANOVA) followed by Tukey’s HSD test (*P < 0.05). All analyses were performed using JMP version 9.0.

## Results

### 22Rv1, DU145, LNCaP, and PC3 are able to grow as self-renewing spheres that can be serially passaged in vitro

**Fig 1:**
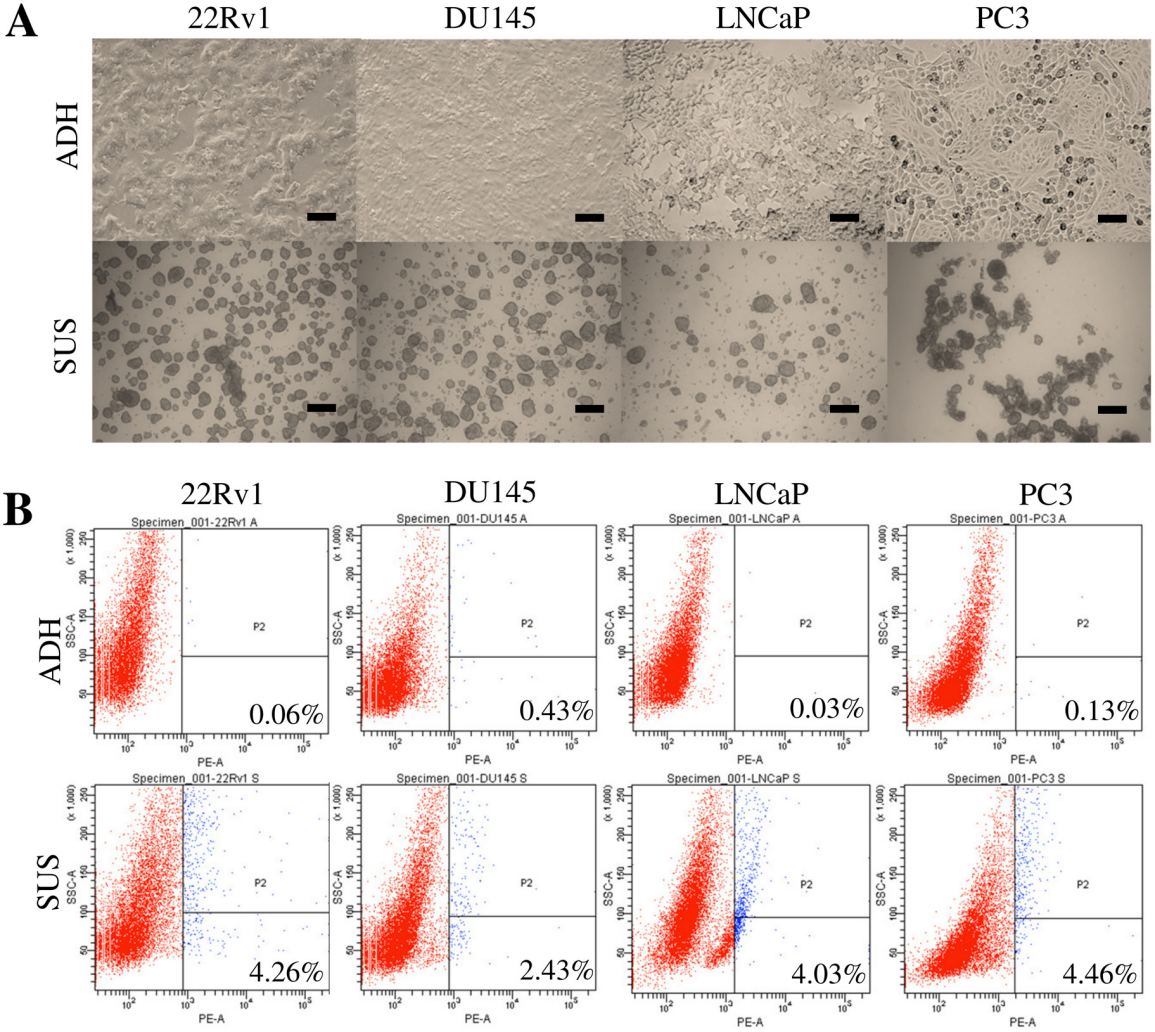
Culture conditions promote the enrichment of CD133^+^ CSCs in prostasphere cultures of PCa cell lines. A) Microscopic examination of parental monolayers (ADH) next to prostasphere-enriched (SUS) cultures. ADH cultures in DMEM-FBS exhibit characteristic epithelial morphology, and are in close contact with one another. SUS cultures in hPCM-PLUS display heterogeneous morphologies, ranging from tight, round-shaped spheres (22Rv1 and DU145) to larger more irregular structures (LNCaP and PC3). Images are representative of 12-day old monolayer and primary sphere cultures. Scale bar = 200 μm. B) Flow cytometric analysis of freshly isolated parental (ADH) and prostasphere (SUS) cells for the identification of CD133^+^ subpopulations. Representative dot plots of PE-labeled CD133^+^ (293C3) cells. Percentages shown correspond to the average of three independent experiments.

**Fig 2:**
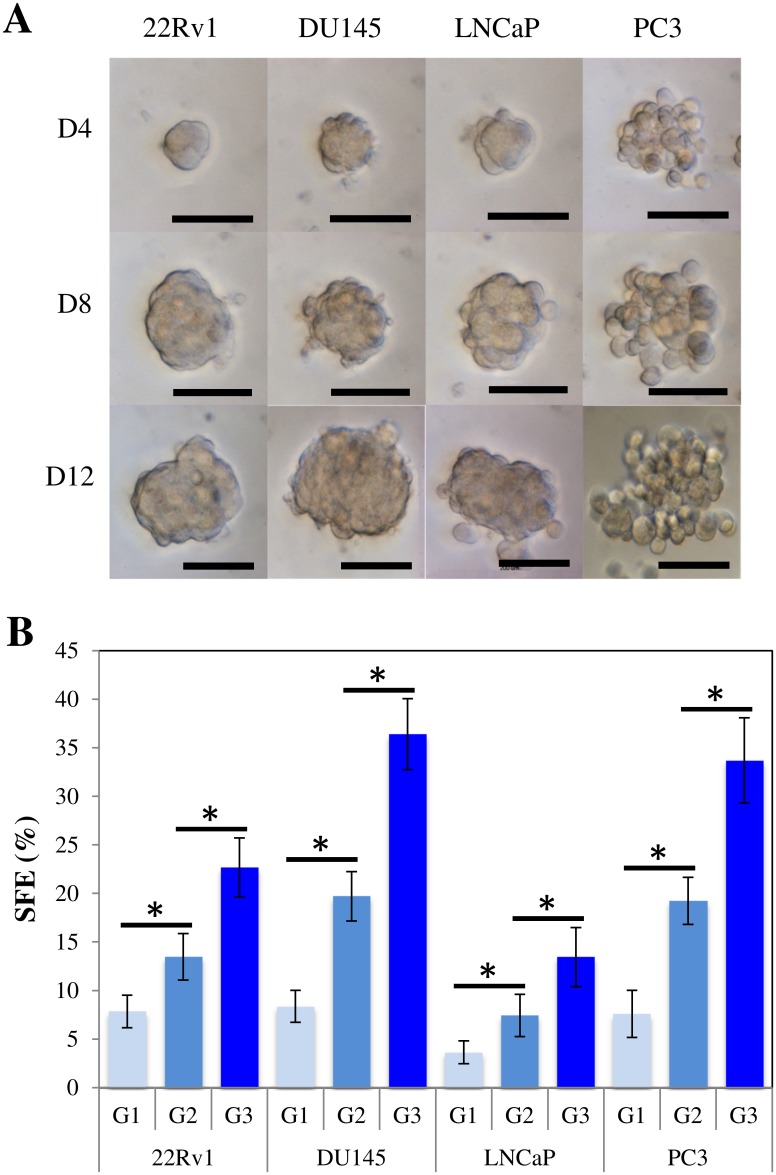
Morphology and Sphere-forming efficiency are cell line dependent. A) Morphological characterization of primary prostaspheres grown in hPCM-PLUS. All PCa cell lines formed discernable spheres by day 4 of culture. 22Rv1 and DU145 spheres consist of smaller and more tightly packed together cells. LNCaP and PC3 cells appear larger and more loosely organized. Scale bar = 100 μm. B) Sphere-forming efficiency (SFE) of prostasphere cultures. PCa cells were seeded at a density of 1x10^3^ cells/ml in 6-well ultra-low attachment plates in hPCM-PLUS and incubated for 10 days. The resulting prostaspheres could be serially passaged in vitro for up to three generations (G1-G3). SFE of spheres increased consistently in all PCa cell lines with each generation. No further amplification was attempted at this point. Three independent experiments were carried out, each one in triplicate. Scale bar = 100 μm. (*p < 0.05).

Tumorsphere forming ability was evaluated using a seeding density of 2x10^4^ cells/ml in 6-well ultra-low attachment plates containing 2 mL hPCM-PLUS medium. All four PCa cell lines formed spherical colonies by day 4 of culture. Parental adherent cultures were propagated simultaneously at a maximum confluence level of 80%. Day 12 tumorspheres and monolayers (Fig 1A) were dissociated and analyzed for the expression of the CSC-associated biomarkers CD133 and CD44. Expression of CD44 was detected heterogeneously at high levels in PC3 and DU145 cells, whereas minimum to no detectable expression was observed in 22Rv1 and LNCaP cells (S1A Fig). Fig 1B shows that CD133 was observed consistently at extremely low levels (< 0.5%) in all cell lines. The panel also shows that in contrast to parental cultures, the percentage of CD133^+^ events increased significantly (*p* < 0.05) in all prostasphere cultures of 22Rv1 (0.067%±0.058% to 4.267%±1.419%), DU145 (0.433%±0.252% to 2.433%±0.709%), LNCaP (0.033%±0.058% to 4.033%±1.966%), and PC3 (0.133%±0.058% to 4.467%±1.358%) cells. Prostaspheres quickly reached diameters > 100 μm by day 12 of culture (Fig 2A). The 22Rv1 and DU145 cells formed compact and round-shaped spheres with well delimited borders, whereas the LNCaP and particularly the PC3 cells formed more irregular structures consisting of larger individual cells. The sphere-forming efficiency (SFE) was evaluated for first- (Day 10), second- (Day 20), and third- (Day 30) generation spheres. SFE invariably increased (*p* < 0.05) with each new generation in all cell lines (Fig 2B). Interestingly, direct observation of PC3 and DU145 cells grown in suspension revealed further budding from prostaspheres at this point (S1B Fig). This observation suggests the potential formation of branching structures from established spheres.

### CD133^+^ prostasphere cells exhibit increased clonogenicity in both adherent and semisolid colony-forming cell (CFC) assays

**Fig 3:**
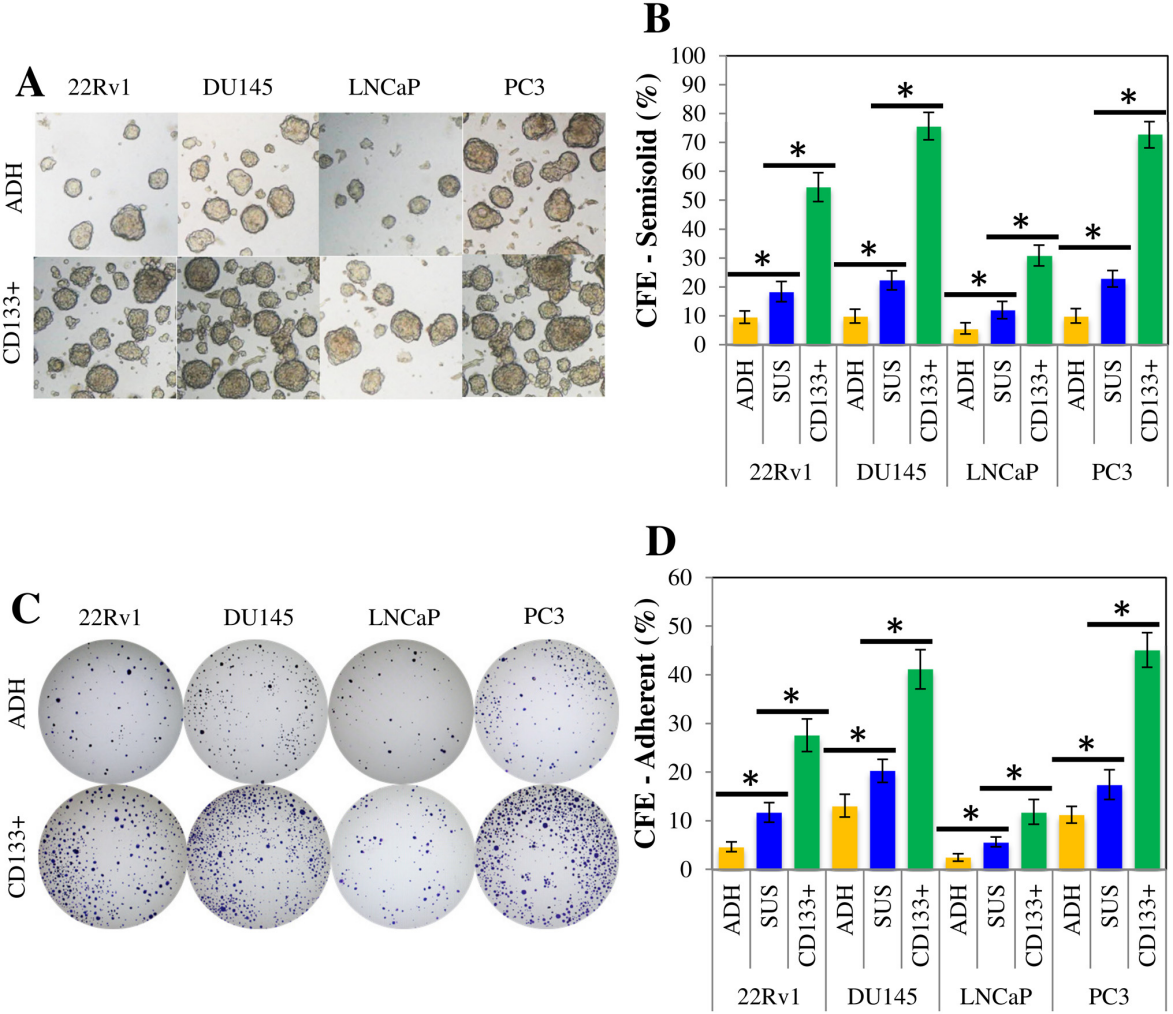
Prostasphere-derived cells become increasingly more clonogenic due to CD133^+^ cells. A) Representative micrographs of suspension colonies from parental (ADH) and CD133^+^ cells. B) Semisolid colony-forming cell (CFC) assay. Parental (ADH) and prostasphere (SUS) cultures were dissociated at day 12 of culture. CD133^+^ cells were isolated via MACS using the 293C3 antibody. 1x10^3^ cells from each condition were seeded in 35-mm petri dishes with StemXVivo methylcellulose concentrate mixed with DMEM-FBS or hPCM-PLUS. Results are expressed as means ± StdDev. (*p < 0.05). C) Representative micrographs of adherent colonies from parental (ADH) and CD133^+^ cells. D) Adherent colony-forming cell (CFC) assay. Cells were processed as described before for semisolid CFC assay. Then, 1x10^3^ cells were seeded in 35-mm petri dishes with DMEM-FBS or hPCM-PLUS. Results are expressed as means ± StdDev. (*p < 0.05).

The colony forming efficiency (CFE) of the parental cultures as well as the prostasphere-derived cells were assayed in parallel in semisolid (Fig 3A) and adherent (Fig 3C) CFC assays. CFE values for monolayer and prostasphere cultures were similar between assays but we observed a consistently higher number of individual colonies in semisolid (Fig 3B) compared to adherent conditions (Fig 3D). All four prostasphere-enriched cell lines displayed higher clonogenicity in both assays compared to their non-enriched counterparts (*p* < 0.05). PC3 cells exhibited the highest clonogenicity for the adherent and semisolid assays. CFE values were consistently higher in CD133^+^ cells (*p* < 0.05) when compared to parental and unsorted prostasphere cells for all cell lines, except for the LNCaP cell line. We were not able to observe a statistically significant difference between CD133^+^ cells and unsorted prostaspheres (*p* < 0.05) in the adherent CFC assay. However, CD133^+^ LNCaP cells did appear to be more clonogenic than unsorted prostaspheres when using the suspension variant of the assay.

### Prostasphere cultures are more resistant to the chemotherapeutic drug Docetaxel

**Fig 4:**
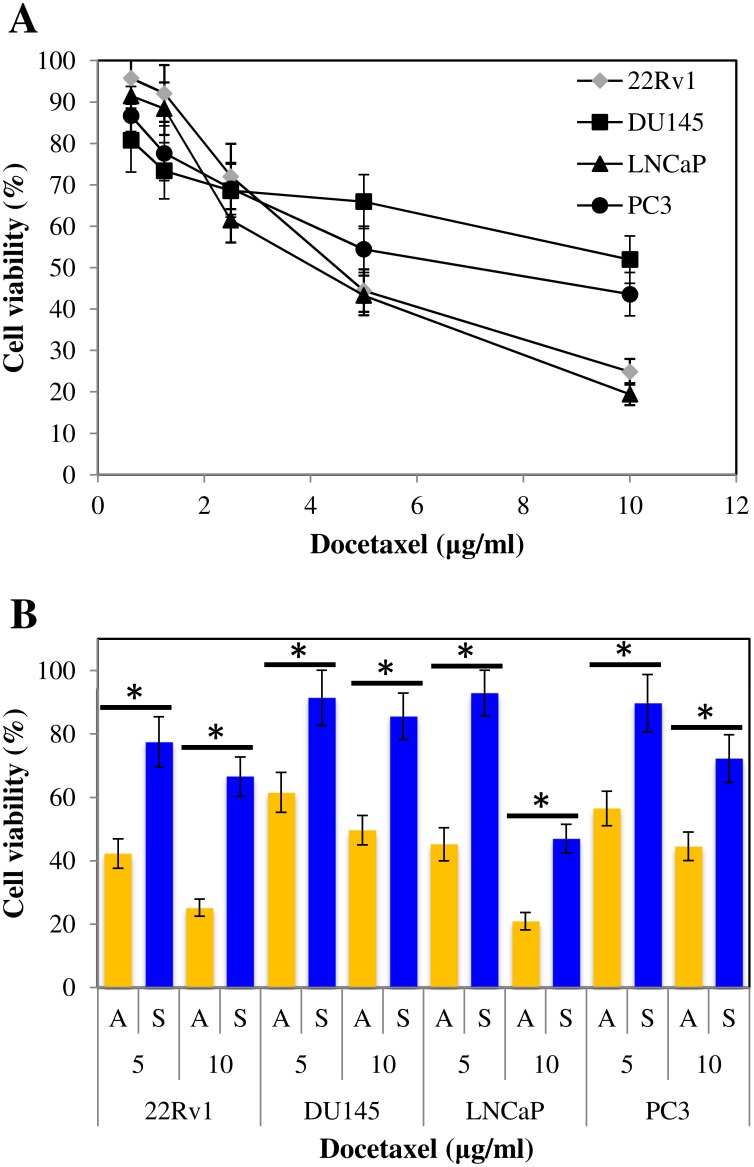
Prostasphere-derived cells are more resistant to the chemotherapeutic drug Docetaxel. A) Establishment of basal sensibility of parental cultures to Docetaxel. 1x10^4^ cells from 12-day old monolayer cultures were incubated with 100 μl DMEM-FBS at a final concentration of 10, 5, 2.5, 1.25, and 0.625 μg/ml Docetaxel. 22Rv1 and LNCaP cells cluster together and exhibit a higher sensibility to the drug compared to DU145 and PC3 cells. Results are expressed as means ± StdDev. B) Evaluation of the chemoresistance of parental and prostasphere cells. 1x10^4^ cells from adherent (A) and prostasphere (S) cultures were incubated with 100 μl DMEM-FBS or hPCM-PLUS media, at a final concentration of 5 and 10 μg/ml Docetaxel. Prostasphere cells display significantly increased chemoresistance to Docetaxel. The figure shows the proportion of viable cells relative to the non-treated control. Results are expressed as means ± StdDev (*p < 0.05).

CSCs are hypothesized to be responsible for PCa resistance to chemotherapeutic drugs. We assessed the basal chemoresistance of the parental monolayer cells by incubating all cell lines with increasing concentrations of Docetaxel (0.625–10 μg/ml) for 24 hours (Fig 4A). Cell viability decreased consistently, but 22Rv1 and LNCaP cells exhibited a higher sensitivity to the drug. We observed a differential response at 5 and 10 μg/ml Docetaxel; therefore, we used these concentrations for further assessment of the chemoresistance of prostaspheres. Chemoresistance to both concentrations of Docetaxel increased significantly (*p* < 0.05) from that observed in the parental cell lines in all tumorsphere cultures (Fig 4B). Prostasphere-derived DU145 cells, in particular, showed the highest cell viability at both concentrations: 85.58%±7.32% at 10 μg/ml, and 91.35%±8.74% at 5 μg/ml. No statistically significant differences were found between CD133-sorted and unsorted prostasphere cultures.

### CD133^+^ prostasphere cells exhibit an increased invasive potential compared to parental monolayer cultures in vitro

**Fig 5:**
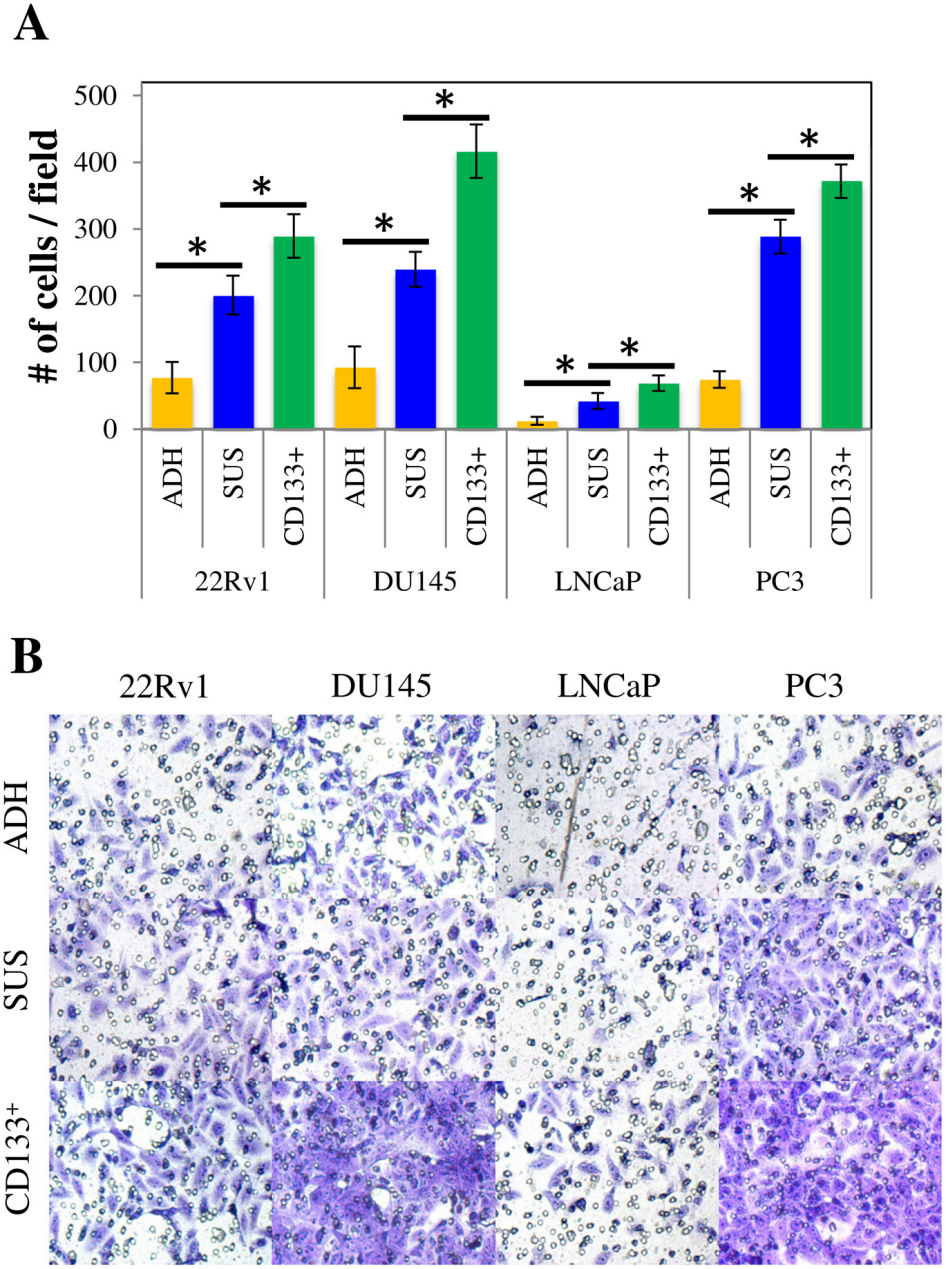
CD133^+^ cells are responsible for the enhanced invasive potential of prostasphere-derived cells. A) Transwell invasion assay. 1x10^5^ parental (ADH), prostasphere (SUS), and CD133^+^ cells were seeded in 200 μl serum-free DMEM-F12 without phenol red on Transwell inserts coated with 50 μl Geltrex LDEV-free reduced growth factor basement membrane matrix. Cells were allowed to invade through the matrix for 24 hours. Results are expressed as means ± StdDev. (*p < 0.05). B) Representative images of Transwell inserts showing invading cells stained with crystal violet solution.

Advanced PCa has a tendency to spread to bone and lymph nodes. Hence, we performed a Transwell invasion assay to determine the metastatic potential of parental and enriched cultures. Following incubation for 48 hours, the numbers of cells that invaded through the Transwell insert were significantly higher for the prostaspheres than for the parental monolayer cells (*p* < 0.05) (Fig 5A). Next, we further examined the role of CD133^+^ cells in the migration of PCa cells and consequently, the establishment of metastatic lesions. The CD133^+^ cells in all cell lines were significantly more invasive than the unsorted prostaspheres (*p* < 0.05). Interestingly, CD133^+^ DU145 cells exhibited the highest invasive potential (416.55±40.161/field). Fig 5B shows representative photomicrographs of random fields with invading cells from adherent, prostasphere, and CD133^+^ cells.

### Identification of overexpressed genes in prostasphere-enriched cultures and CD133^+^ cells

**Fig 6:**
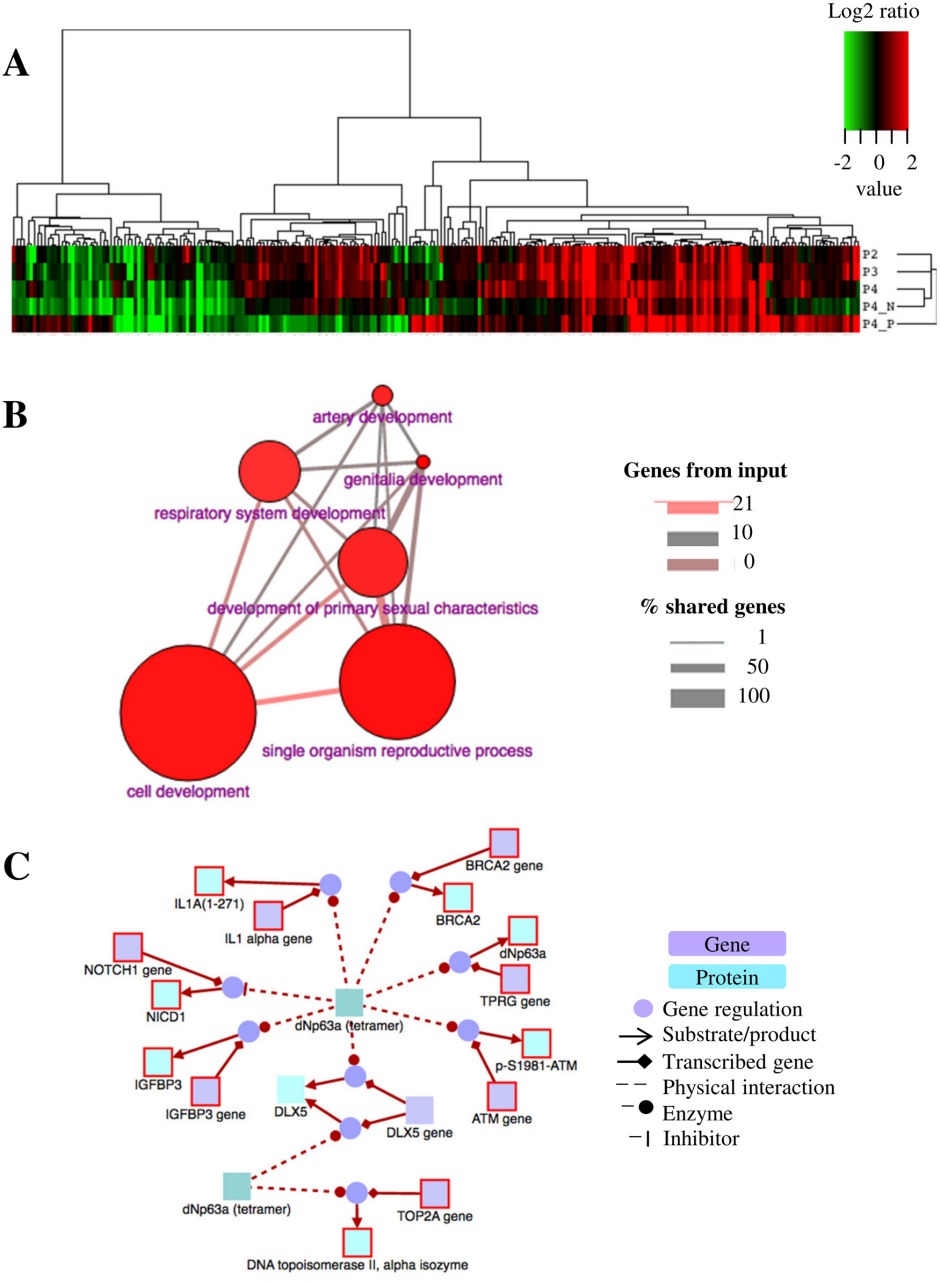
Gene expression profiling and functional annotation analysis: Activation of developmental pathways modulated by ΔNp63α drives the CSC-enrichment observed in prostasphere cultures. A) Heat map showing fold changes in gene expression from prostaspheres at day 4 (P2), day 8 (P3), day 12 (P4), and isolated CD133^-^ (P4_N) and CD133^+^ (P4_P) fractions relative to parental cultures on day 0. Values correspond to the Log2 transformed ratios of the means (n = 3). The figure shows the behavior of the 244 genes assayed throughout the time the cells are kept in culture. B) Gene Ontology (GO) analysis of upregulated transcripts in CD133^+^ cells. GO Biological processes enriched in CD133^+^ cells are all related to developmental pathways (p* < 0.01). The edge width reflects the relative overlap between the nodes (% shared genes). The edge color encodes the number of shared members between the whole GO category and the user-defined background (CodeSets). Sphere size is relative to the number of genes associated with that category. C) Molecular interactions of the transcriptional regulator ΔNp63α with upregulated genes detected in CD133^+^ cells. *ATM*, *TP63*, *IGFBP3*, *NOTCH1*, *BRCA2*, *IL1A* and *TOP2* genes are upregulated in CD133^+^ cells and appear to mediate the downstream activation of developmental molecular pathways. The ΔNp63α tetramer shown at the center of the figure is known to physically interact with these genes. Pathway source: Pathway interaction database (http://pid.nci.nih.gov).

Gene expression profiling of cancer tissues has the potential to improve current diagnostic and prognostic classifications, and to reveal insight into the underlying CSC pathophysiology. Here, we aimed to identify overexpressed genes in magnetically sorted and unsorted PC3 prostasphere cells, compared to parental monolayer cultures. Gene expression data was submitted to the Gene Expression Omnibus repository from NCBI and can be accessed at the GEO website (http://www.ncbi.nlm.nih.gov/geo/) under the GSE67248 accession number. Table 1 lists upregulated transcripts (ratio > 1.5) in CD133+ sorted and unsorted prostasphere cells (See Table A in S1 File for the full set of data). Fig 6A shows the hierarchical clustering of the relative fold changes in the 244 genes analyzed, throughout the 12 days of culture. This graphical representation shows how culture conditions induce global changes in gene expression through time. Functional annotation clustering of upregulated genes with similar biological function included: proteins containing predicted glycosylation sites and sequences targeting them to the secretory pathway, membrane-associated proteins, proteins that participate in cell adhesion and cell surface structure organization, and proteins with tyrosine kinase activity.

Table 2 lists annotation clusters with enrichment factors > 1.3. We also observed evidence for processes such as: response to hormone stimulus, ion homeostasis, protein post-translational modification, positive regulation of cell motility and migration, proteolytic activity, and negative regulation of apoptosis, as well as immunoglobulin-like domains and several proteins related to embryonic organ morphogenesis (See Table A in S2 File for the full list). Overrepresentation analysis of upregulated genes in CD133^+^ cells yielded six enriched gene ontology (GO)-based sets: genitalia development, single organism reproductive process, cell development, development of primary sexual characteristics, artery development, and respiratory system development (*p* < 0.01, Fig 6B). Lastly, our analysis revealed one enriched pathway-based set corresponding to the validated transcriptional targets of the transcriptional regulator ΔNp63α (*p* < 0.05, Fig 6C).

**Table 2:** Functional annotation clustering of upregulated genes in CD133^+^ sorted and unsorted prostasphere cells.

Term	p-value	Genes
**Prostasphere cells vs. parental cells**		
**Enrichment Score: 2**		
Transmembrane	0.0076	*EGFR*, *ERBB2*, *ERBB4*, *FGFR2*, *IFNGR1*, *FAS*, *ITGB1*, *MCL1*, *MMP14*, *MPL*, *MUC1*, *NGFR*, *NOTCH3*, *PDGFRB*, *ABCB1*, *BCL2*, *CAV1*, *TNFSF10*, *CD34*
**Enrichment Score: 1.63**		
Signal	0.0208	*CTGF*, *EGFR*, *ERBB2*, *ERBB4*, *FGFR2*, *FOLR1*, *FRZB*, *GAS1*, *IFNGR1*, *IGFBP3*, *FAS*, *ITGB1*, *CXCL9*, *MMP2*, *MMP9*, *MMP14*, *MPL*, *MUC1*, *NGFR*, *NOTCH3*, *PDGFA*, *PDGFRB*, *PLA2G2A*, *TIMP3*, *CD34*
Disulfide bond	0.0279	*CTGF*, *EGFR*, *ERBB2*, *ERBB4*, *FGFR2*, *FOLR1*, *FRZB*, *IFNGR1*, *IGFBP3*, *FAS*, *ITGB1*, *CXCL9*, *MMP2*, *MMP9*, *MMP14*, *MPL*, *NGFR*, *NOTCH3*, *PDGFA*, *PDGFRB*, *PLA2G2A*, *TIMP3*,
**CD133^+^ vs. CD133^-^ prostasphere cells**		
**Enrichment Score: 2.4**		
Glycoprotein	0.0016	*CDH11*, *WFDC2*, *COL1A1*, *CSF3*, *EGFR*, *ERBB2*, *ERBB3*, *ESR1*, *FAT1*, *FGFR1*, *FGFR4*, *FRZB*, *HMMR*, *IGFBP3*, *KLK3*, *IL1A*, *IL6*, *KDR*, *L1CAM*, *LAMB1*, *LIF*, *MET*, *MMP2*, *MSTR1*, *MUC1*, *NOTCH1*, *NOTCH3*, *SERPINE1*, *PDGFRA*, *PDGFRB*, *PLAT*, *PTGS2*, *PTK7*, *PTPRG*, *SHH*, *TYR03*, *WNT10B*, *PROM1*
Signal	0.0157	*CDH11*, *WFDC2*, *COL1A1*, *CSF3*, *EGFR*, *ERBB2*, *ERBB3*, *FAT1*, *FGFR1*, *FGFR4*, *FRZB*, *IGFBP3*, *KLK3*, *IL6*, *KDR*, *L1CAM*, *LAMB1*, *LIF*, *MET*, *MMP2*, *MSTR1*, *MUC1*, *NOTCH1*, *NOTCH3*, *SERPINE1*, *PDGFRA*, *PDGFRB*, *PLAT*, *PTGS2*, *PTK7*, *PTPRG*, *SHH*, *TYR03*, *WNT10B*, *PROM1*
**CD133^+^ prostasphere cells vs. parental cells**		
**Enrichment Score: 2.47**		
Glycoprotein	0.0028	*CDH11*, *COL1A1*, *CTGF*, *EGF*, *EGFR*, *ERBB2*, *ERBB3*, *ERBB4*, *ESR1*, *FAT1*, *FGFR1*, *FGFR2*, *FGFR4*, *FOLR1*, *GAS1*, *GNAS*, *IGFBP2*, *IGFBP3*, *FAS*, *ITGB1*, *KDR*, *L1CAM*, *LAMB1*, *MET*, *MMP9*, *MSTR1*, *MUC1*, *NOTCH1*, *NOTCH3*, *SERPINE1*, *PDGFRA*, *PDGFRB*, *PLAT*, *WNT4*, *PTK7*, *PTPRG*, *TGFBR2*, *TYR03*, *WNT10B*, *CD34*, *CDH1*
Signal	0.0049	*CDH11*, *COL1A1*, *CTGF*, *EGF*, *EGFR*, *ERBB2*, *ERBB3*, *ERBB4*, *FAT1*, *FGFR1*, *FGFR2*, *FGFR4*, *FOLR1*, *GAS1*, *IGFBP2*, *IGFBP3*, *FAS*, *ITGB1*, *KDR*, *L1CAM*, *LAMB1*, *MET*, *CXCL9*, *MMP9*, *MSTR1*, *MUC1*, *NOTCH1*, *NOTCH3*, *SERPINE1*, *PDGFRA*, *PDGFRB*, *PLA2G2A*, *PLAT*, *WNT4*, *PTK7*, *PTPRG*, *TGFBR2*, *TYR03*, *WNT10B*, *CD34*
**Enrichment Score: 2.09**		
Transmembrane	0.0040	*CDH11*, *EGF*, *EGFR*, *ERBB2*, *ERBB3*, *ERBB4*, *FAT1*, *FGFR1*, *FGFR2*, *FGFR4*, *FAS*, *ITGB1*, *KDR*, *L1CAM*, *MCL1*, *MET*, *MST1R*, *MUC1*, *NOTCH1*, *NOTCH3*, *PDGFRA*, *PDGFRB*, *PTK7*, *PTPRG*, *BCL2*, *TGFBR2*, *TYR03*, *CD34*, *CDH1*
**Enrichment Score: 1.75**		
Cell adhesion	0.0054	*CDH11*, *CTGF*, *CTNNB1*, *FAT1*, *ABL1*, *ITGB1*, *L1CAM*, *LAMB1*, *PTK7*, *TYR03*, *CD34*, *CDH1*
**Enrichment Score: 1.42**		
Tyrosine-protein kinase	0.0756	*CSK*, *EGFR*, *ERBB2*, *ERBB3*, *ERBB4*, *FGFR1*, *FGFR2*, *FGFR4*, *ABL1*, *FYN*, *KDR*, *MET*, *MSTR1*, *PDGFRA*, *PDGFRB*, *TYR03*

Functional annotation was carried out using the Swiss-Prot and Protein Information Resource Keywords (SP_PIR_Keywords) database from DAVID Bioresources. Background was defined as the 244 genes contained in both CodeSets. Clusters with an enrichment score > 1.3 are shown. The total number of clusters identified was: 61 (prostasphere vs. parental), 60 (CD133+ vs. CD133-), and 34 (CD133+ vs. parental) (Shown in Table A in S1 File).

## Discussion

PCa has been postulated to involve alterations in the balance between epithelial cell division and differentiation, resulting from aberrant SC growth [21]. Similar to normal adult SCs, malignant CSCs are able to self-renew and generate further differentiated cells that constitute the bulk of the tumor. These CSCs are known to exhibit increased chemoresistance, invasiveness, and clonogenicity, as well as specific gene signatures that distinguish them from the rest of the cells in a tumor. Understanding the molecular pathways that drive CSC survival will help elucidate the mechanisms underlying PCa physiopathology. Therefore, there is an urgent need for experimental platforms that take into account the critical role of CSCs in PCa. Previous findings have demonstrated that stem-like populations persist in commercial PCa cell lines such as, 22Rv1, DU145 [12], LNCaP, and PC3 [22]. However, these cells are often present at extremely low levels, which diminishes their experimental potential.

### Culture conditions induce CSC-enrichment and determine cellular fate in prostasphere cultures

We demonstrated the enrichment of a CD133^+^ CSC-like subpopulation in four commercial PCa cell lines (22Rv1, DU145, LNCaP, and PC3) (Fig 1). The heterogeneous expression of the CD44 marker led us to use CD133/2 (293C3) as the only selection criterion to denote CSC-like populations. Although tumorsphere culture has been demonstrated to be an effective strategy for progenitor enrichment in cell lines, the optimization of culture conditions that could be applied to various cell lines is still a work in progress. Fan et al. demonstrated efficient formation of PC3 tumorspheres using serum-free DMEM-F12 supplemented with 20 ng/ml EGF [22]. The PC3 cells formed spheres that could be serially subcultured, but the LNCaP cells were unable to do so. Wang et al. were also unable to obtain viable spheres from LNCaP cells when using a similar DMEM-F12 formulation, further supplemented with human leukemia inhibitory factor [23]. Concordant to this, LNCaP as well as 22vR1 cells were unable to form spheres when cultured in our DMEM-PLUS formulation (S1C Fig), whereas PC3 and DU145 cells readily formed spheres. In contrast, Duhagon et al. were able to grow LNCaP spheres using a similar serum-free DMEM-F12 formulation further supplemented with KnockOut Serum Replacement (Invitrogen), and 10 ng/mL bFGF [24]. In this contribution we were able to obtain highly proliferative spheres from all four PCa cell lines using our hPCM-PLUS formulation. The numbers of generated spheres and sphere morphology were cell-line dependent (Fig 2A), but the self-renewing capacity of prostaspheres increased in all cases with each successive generation (Fig 2B)—a distinct hallmark of CSC self-renewal. These contrasting results demonstrate the substantial impact of seemingly subtle changes in culture conditions on the final fate of cells propagated in vitro.

The enriched progenitor identity of prostasphere-derived cells was also supported by their increased proliferative, chemoresistant, and invasive potential. We observed a higher number of colony-forming cells in both monolayer and suspension CFCs, compared to parental cultures (Fig 3). Magnetically sorted CD133^+^ cells were consistently more clonogenic in all cases, which demonstrates the critical role of these cells driving malignant cell proliferation and tumor growth. Prostasphere cells were also more resistant to the chemotherapeutic drug Docetaxel (Fig 4) and were more invasive (Fig 5) than parental cultures. These results agree with previous literature indicating that Docetaxel-resistant DU145 and PC3 cells express molecular markers associated with stem-like populations [25]. Moreover, similar to what we observed in our CFCs assays, CD133^+^ cells exhibited a significantly higher chemoresistant and invasive potential, when compared to parental monolayer cultures. Taken together, these findings suggest that the enriched subpopulation, represented by the CD133/2 (293C3) antigen, is directly involved in the establishment of the phenotypic determinants of CS-like cells within these cell lines.

### Gene expression profiling of prostaspheres reveals the upregulation of genes that contribute to the CSC phenotype

Previous groups have described the implementation of different technologies for profiling gene expression patterns related to PCa [26–29]. Currently, the use of new high-throughput methods of gene expression analysis is improving our understanding of PCa physiopathology. Here, we used the nCounter system from NanoString Technologies to analyze the expression of 244 cancer-related genes in PC3 prostaspheres, as well as isolated CD133^+^ fractions. Gene expression profiling of enriched cultures revealed several significantly upregulated genes in CD133^+^-sorted and unsorted prostasphere cells (Table 1). The most highly overexpressed genes (fold change > 4) are discussed individually since they represent the most promising targets for CSC-specific intervention revealed in this particular study. First, we compared the upregulated transcripts in the CD133^+^ fraction relative to the CD133^-^ and focused on *CSF3*, *TERT*, *KLK3*, and *IL1A*:

***CSF3*** (20.16 fold change): Colony-stimulating factor 3, also known as granulocyte colony-stimulating factor (G-CSF), is a glycoprotein that modulates the production, differentiation, and function of granulocytes, as well as the mobilization of hematopoietic progenitor cells from the bone marrow [18]. Recent evidence suggest that G-CSF also has a role in promoting tumor neurogenesis and PCa progression, making it an attractive target for therapy [30].

***TERT*** (7.77 fold change): The telomerase reverse transcriptase constitutes the catalytic component of the telomerase holoenzyme complex that is essential for the replication of eukaryotic chromosome termini [18]. Erosion of telomeric sequences constitutes the basis of the cellular clock that eventually instructs the cell to undergo senescence. Mutations leading to overexpression of *TERT* enhance the invasiveness of tumor cells, are more prevalent in older patients with invasive diseases and advanced tumor stages, and are associated with significantly shorter patient survival time [31].

***KLK3*** (7.23 fold change): Kallikrein 3, also known as prostatic specific antigen (PSA), has been used traditionally as a biomarker for the diagnosis and monitoring of PCa [18]. Nevertheless, the precise role of its serine-type peptidase activity in the pathophysiology of PCa remains unclear. Interestingly, PC3 cells are known to lack expression of PSA [32]; nevertheless, emerging evidence suggests a critical role for prostate-specific kallikreins (human kallikrein 2 and PSA) in the promotion of cancer cell growth through pro-angiogenic stimulation [33]. Furthermore, expression of *KLK3* and *KLK4* leads to changes that appear similar to the epithelial-to-mesenchymal transition (EMT) when expressed in PC3 cells [34]. Taken together, these findings suggest that PSA may play a role in CSCs from prostate tumors that exhibit a similar phenotype to the PC3 cell line.

***IL1A*** (5.1 fold change): Inflammation has been traditionally considered an important etiological factor in PCa. An involvement of interleukin-1 α, one of the major pro-inflammatory cytokines, is also postulated in the development of metastatic PCa. Metastasis to the bone is observed in more than 80% of PCa cases [35]. Recently, IL-1 alpha was demonstrated to induce an immunosuppressive response in mesenchymal SCs, which in turn promoted the growth of PCa cells [36]. The production of IL-1 alpha by tumor-associated macrophages also promotes angiogenesis and tumor growth in murine animal models [37].

Next, we compared the upregulated transcripts in CD133^+^ cells relative to parental cultures and focused on *IGFBP3*, *CSF3*, *IL1A*, *BCL2A1* and *EGR1* (*IGFBP3*, *CSF3* and *IL1A* were also upregulated relative to CD133^-^ cells and are already discussed in the previous section).

***BCL2A1*** (4.11 fold-change): B-cell lymphoma 2 (BCL2) proteins are important cell death regulators [18]. Many of these proteins act as cellular oncogenes that not only promote tumorigenesis but also contribute to resistance to chemotherapeutic drugs and failure of anti-cancer treatments [38]. Bcl-2-related protein A1 is considered a potential therapeutic target in B-cell malignancies. Brien *et al*. demonstrated that the expression of certain peptide aptamers of the Bcl-2 related protein A1 sensitize B-cell lines to apoptosis induced by chemotherapeutic agents [39]. Particularly in prostate tissues, overexpression of the transcription factor C/EBP in DU145 and PC3 cells significantly upregulated promoter activities of metastatic and anti-apoptotic genes, including *BCL2A1* [40]. Despite this evidence, the role of *BCL2A1* in prostate CSCs has not been fully explored.

***EGR1*** (4 fold change): The early growth response gene is a transcriptional regulator that is frequently overexpressed inPCa [18]. The gene expression pattern of *EGR1* suggests that it could potentially regulate a number of steps involved in the initiation and progression of prostate cancer, such as mitogenesis, invasiveness, angiogenesis, and metastasis [41]. RT-PCR and western blot analysis have demonstrated that EGR-1 exhibits higher expression levels in PCa cells with an androgen independent phenotype, such as PC3 [42]. Concordant to this, small interfering RNA (siRNA) silencing of *EGR1* is able to induce apoptosis and inhibit growth of PC3 cells [43]. The induction of *EGR1* by external stimuli is generally transient but appears to be sustained in PCa [44]. Moreover, expression levels of EGR-1 increase with the degree of malignancy, as measured by the Gleason score of the tumor.

Finally, we compared the upregulated transcripts in prostaspheres relative to parental cells and focused on *GAS1* and *IGFBP3*:

***GAS1*** (5.36 fold change): Growth arrest-specific 1 is a putative tumor suppressor protein that blocks entry to S phase and prevents cycling of normal and malignant cells [18]. Recent studies have demonstrated that a soluble form of GAS-1 reduces the vascularization of xenotransplanted tumors by preventing the migration of endothelial cells [45].

***IGFBP3*** (5.24 fold change): is a member of the insulin-like growth factor (IGF) binding protein family, a group of proteins that is known to prolong the half-life of IGFs and to alter their interaction with their respective cell surface receptors [18]. Similarly to GAS-1, IBP-3 (the protein coded by the IGFBP3 gene) behaves as a pro-apoptotic, anti-metastatic, and anti-angiogenic protein [46].

Despite the anti-cancer roles reported for both of these proteins, it is important to note that their molecular function may vary depending on their cellular context [47]. Spagnuolo et al. reported that GAS-1 does not inhibit the growth of vascular endothelial cells but that its expression strongly protects the cells from apoptosis [47]. Other groups have also demonstrated different roles for GAS-1 in modulating chondrocyte differentiation [48] and promoting cell proliferation in the central nervous system [49]. Similar to what has been reported for GAS-1, immunohistochemical analysis has revealed elevated levels of IBP-3 in the hyperplastic fibromuscular stroma of benign prostatic hyperplasia (BPH) specimens and in the tumor-adjacent stroma of high-grade PCa [50]. This means that IBP-3 may also play a role in fibroblast-to-myofibroblast differentiation, a hallmark of stromal remodeling associated with PCa and BPH.

### Functional annotation analysis reveals molecular pathways that could be responsible for the increased CSC identity of prostasphere cultures

Although individual analysis of top upregulated genes could pinpoint specific targets for therapeutic intervention, this approach fails to take into consideration the intricate molecular networks underlying complex cellular processes such as proliferation, chemoresistance and invasion. Therefore, we analyzed our gene expression data using the functional annotation tool from DAVID Bioinformatics resources [19]. Functional annotation revealed several overrepresented gene clusters that could be responsible for the phenotypic behavior of our prostasphere cultures. The top functional categories identified by DAVID in CD133^+^ cells were related to membrane-associated proteins participating in cell adhesion or exhibiting protein tyrosine-kinase activity (Table 2, full list in Table A in S2 File).

Based on our results we conclude that the increased proliferative and clonogenic potential observed in vitro could be driven by positive regulators of epithelial cell proliferation, such as *ERBB4*, *ERBB2*, *GAS1*, and *EGFR*, through EGF signaling. Increased expression of EGFR is associated with disease relapse and transformation to an androgen independent state [51]. Negative regulation of apoptosis is also an important feature of CSCs. BCL2 proteins are central regulators of caspase activation, and play a key role in cell death by regulating the integrity of the mitochondrial and endoplasmic reticulum membranes [52]. Our results suggest that these cells could evade apoptosis through signaling that involves the BCL2 family of proteins, such as Bcl-2 (coded by the BCL2 gene), Bcl-2 related protein A1 (coded by the BCL2A1 gene), and Mcl-1 (Coded by the MCL1 gene). *BCL2A1* in particular was upregulated in CD133^+^ cells relative to CD133^-^ and parental cells (Table 1). This observation suggests a direct involvement of *BCL2A1* in the survival of malignant prostate CSCs.

Increased tumor cell migration and invasion were also observed in vitro. Positive regulators of cell migration that were upregulated in these cells included: *EGFR*, *ERBB4*, *FGF2*, *KDR*, *LAMB1*, *MMP9*, *PDGFA*, *PDGFRA*, *PDGFRB*, and *BCL2*. Interestingly, Petz et al. have demonstrated that PDGF enhances translation of the *LAMB1* gene, a known regulator of tumor cell migration and invasion [53]. Similar to *BCL2A1*, *PDGFRA* was also upregulated in CD133^+^ cells and is heavily associated with skeletal metastases [18]. Recent evidence suggests an important role for the PDGF subunit A in facilitating the initial lodging and subsequent progression of PCa cells within the bone microenvironment [35]. Furthermore, it has been demonstrated that antibody blockage of the PDGF subunit A reduces the size of established skeletal metastases [54]. These observations suggest that PDGFRA signaling is directly involved in the ability of prostate CSCs to establish metastatic lesions. Moreover, expression of the matrix metalloproteinases coded by the *MMP2*, *MMP9*, and *MMP14* genes confers these cells the ability to degrade extracellular matrix proteins, an important step in various stages of cancer progression, including angiogenesis, invasiveness, and metastasis [55].

These results demonstrate that patient-specific and multi-targeted therapies that are directed specifically against CSCs could be designed using in vitro culture systems coupled with high-throughput gene expression profiling tools.

### CSC enrichment in prostasphere cultures correlates with activation of development-related molecular pathways

Gene ontology analysis was carried out using ConsensusPathDB and the upregulated genes in CD133^+^ cells, relative to the CD133^-^ fraction. Interestingly, overrepresentation analysis yielded six enriched gene ontology-based sets related to cell development. Furthermore, our analysis revealed an overrepresentation of the enriched pathway-based set corresponding to the validated transcriptional targets of ΔNp63α isoforms (*p* = 0.005). ΔNp63α is an epithelial progenitor cell marker that maintains epidermal stem cell self-renewal capacity [56]. Previous reports have demonstrated that ΔNP63α induces EMT in primary human keratinocytes [57], and that increases the percentage of CSCs in MCF7 cells can increase, which leads to increased cancer cell proliferation, clonogenicity, anchorage-independent growth, drug resistance, and incidence of xenografted tumors in vivo [58]. More recently, Pignon et al. reported that ΔNp63α^+^ cells of the urogenital sinus are able to generate all epithelial lineages of the prostate and bladder [59], further confirming the role of ΔNp63α^+^ cells as the stem/progenitor population in these type of epithelia during development. Taken together, our results suggest that the CSC enrichment observed in our prostasphere cultures occurs through ΔNp63α-mediated activation of stem/development-related genes. Interestingly, this radical shift in gene expression patterns towards a more “stem-like” state is achieved simply by modifying culture conditions, without the need for exogenous DNA insertion or direct manipulation of the genome.

## Conclusion

Biopsy samples obtained from different patients, and even from different zones within the same tumor can vary significantly in terms of their genetic and phenotypic characteristics. We propose that the wide selection of CCLs available can be used to circumvent this type of issues, since they can be representative of tumors of diverse clinico-pathological origins. Furthermore, in vitro enrichment strategies can be used to increase the number of stem/progenitor cells contained in CCLs, making them an efficient alternative to the highly restricted human biopsy samples.

In this contribution, we selected four different and widely used PCa cell lines (22Rv1, DU145, LNCaP, and PC3) of various tissue origins as experimental models. These CCLs are representative of prostate tumors that correspond to various clinical stages of the disease. For example, LNCaP cells are androgen-dependent and show a relatively indolent behavior, similar to the vast majority of prostate tumors encountered clinically. On the other hand, PC3 cells are androgen-independent and exhibit a highly aggressive behavior concordant with that of hormone-refractory PCa. All of these cell lines became significantly more clonogenic, chemoresistant, and invasive when propagated in our culture protocol, phenotypic characteristics associated with CSCs in vivo. Therefore, the intrinsic differences in these cell lines allow the extrapolation of our findings to different clinical scenarios.

We propose that culture systems with the ability to maintain, restore, or even enrich the cancer stem-like compartment from tissues with extremely scarce progenitor populations could be implemented clinically in order to assay actual samples from patients undergoing treatment. Genetic and phenotypic characteristics could then be analyzed and readily interpreted in order to present the patient with a more accurate diagnostic and/or prognostic scenario.

## Supporting Information

S1 Fig Expression of the CD44 biomarker and morphology of 12-day-old PC3 and DU145 prostasphere cultures. A) Flow cytometric analysis of freshly isolated PC3 and DU145 prostaspheres for the identification of CD44^+^ subpopulations. The figure shows representative dot plots of PE/CY7-labeled CD44^+^ cells in PC3 and DU145 cells. Expression of the CD44 biomarker was observed at high levels in these cells. No measurable expression of CD44 was detected in LNCaP or 22Rv1 in our experiments. B) Microscopic examination of 12-day-old PC3 and DU145 prostasphere cultures. PC3 and DU145 cells exhibit further budding from established spheres (white arrows). Complex branching structures become apparent at this point in culture. Scale bar = 100 μm.
(TIFF)

S1 File Table A. Gene expression profiling of PC3 cells grown as prostaspheres in hPCM-PLUS culture medium. Cultures were sampled on day 0 (EPI_1), day 4 (EPI_2), day 8 (EPI_3), and day 12 (EPI_4). Day 12 spheres were also dissociated and separated using MACS and the Anti-CD133/2 (293C3)-PE into CD133+ (EPI_P) and CD133- (EPI_N) fractions. Total RNA was extracted and purified from these fractions, as well as from magnetically. Gene expression profiling was carried out using the Cancer Reference and ITESM panels from NanoString Technologies. Results are shown as normalized counts from three independent experiments.
(XLSX)

S2 File Table A. Functional annotation clustering of upregulated genes in prostasphere and CD133^+^ cells. Table lists the complete set of enriched clusters identified using the functional annotation tool from DAVID. Stringency was set as “high”. %: Percentage of gene overlapping between user’s input and the whole category; P: p-value for the significance of gene-term enrichment calculated with a modified Fisher's Exact Test (EASE Score). Fold: Fold enrichment relative to the background, defined as the 244 genes contained in both CodeSets.
(XLSX)
