# Supplementary figures and images for: Enrichment of the Cancer Stem Phenotype in Sphere Cultures of Prostate Cancer Cell Lines Occurs through Activation of Developmental Pathways Mediated by the Transcriptional Regulator ΔNp63α
Source: PLoS One. 2015 Jun 25;10(6):e0130118. doi: 10.1371/journal.pone.0130118 (PMC4481544; doi:10.1371/journal.pone.0130118)

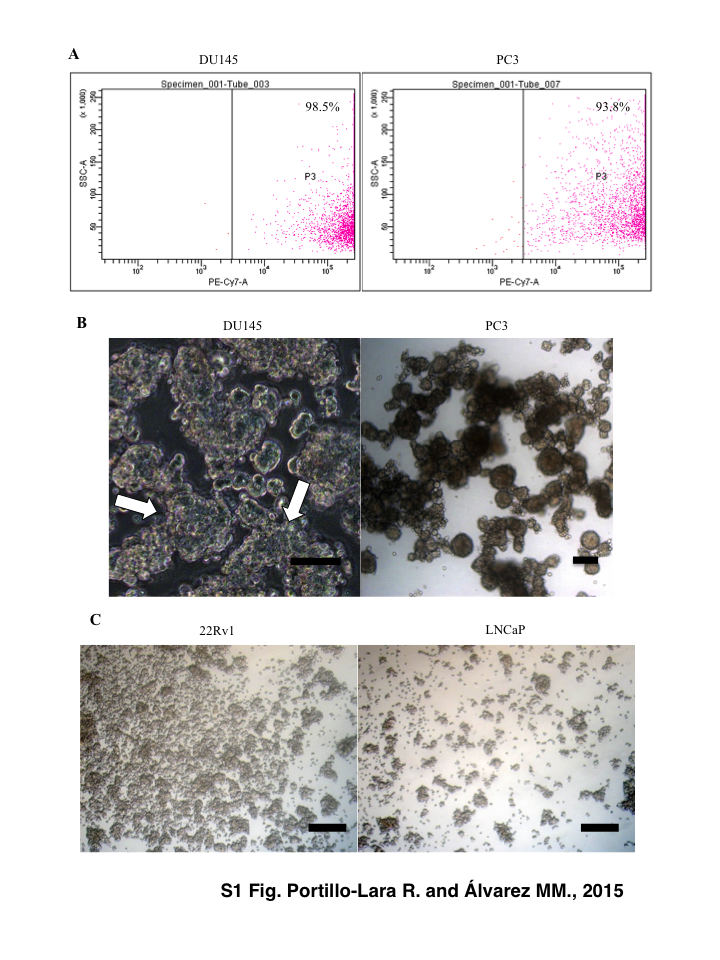

Supplement: S1 Fig — A) Flow cytometric analysis of freshly isolated PC3 and DU145 prostaspheres for the identification of CD44+ subpopulations. The figure shows representative dot plots of PE/CY7-labeled CD44+ cells in PC3 and DU145 cells. Expression of the CD44 biomarker was observed at high levels in these cells. No measurable expression of CD44 was detected in LNCaP or 22Rv1 in our experiments. B) Microscopic examination of 12-day-old PC3 and DU145 prostasphere cultures. PC3 and DU145 cells exhibit further budding from established spheres (white arrows). Complex branching structures become apparent at this point in culture. Scale bar = 100 μm. (TIFF) [file pone.0130118.s001.tiff]
